# COVID-19 Crisis in Brazil: Post-Vaccination Seroprevalence and Infection in More and Less Vaccinated Municipalities in a Northeastern State

**DOI:** 10.3390/life14010094

**Published:** 2024-01-08

**Authors:** Ronaldy Santana Santos, Marina dos Santos Barreto, Eloia Emanuelly Dias Silva, Beatriz Soares da Silva, Pamela Chaves de Jesus, Deise Maria Rego Rodrigues Silva, Pedro Henrique Macedo Moura, Jessiane Bispo de Souza, Leticia Milena Machado dos Santos, Adriana Gibara Guimarães, Lucas Alves da Mota Santana, Lysandro Pinto Borges

**Affiliations:** 1 Department of Pharmacy, Federal University of Sergipe, São Cristóvão 49100-000, SE, Brazil; sbarretomarina@outlook.com (M.d.S.B.); eloiaemanuelly@gmail.com (E.E.D.S.); biasas@hotmail.com (B.S.d.S.); pamcjesus@outlook.com (P.C.d.J.); deisemaria26@academico.ufs.br (D.M.R.R.S.); phmm694@gmail.com (P.H.M.M.); jeisse.nik@hotmail.com (J.B.d.S.); leticiammachado54@gmail.com (L.M.M.d.S.); adrianagibara@hotmail.com (A.G.G.); 2 Graduate Program in Dentistry, Federal University of Sergipe, São Cristóvão 49100-000, SE, Brazil; lucassantana.pat@gmail.com

**Keywords:** vaccination, SARS-CoV-2, infection, municipalities, Brazil

## Abstract

Background: Given the impact of the pandemic in Brazil, vaccination is essential to prevent illness and death. Thus, this study sought to compare, after vaccination, the circulation of SARS-CoV-2 and the response to vaccination in the least and most vaccinated municipalities of a Brazilian state during the height of the pandemic when the Omicron variant was dominant. Methods: We tested for the SARS-CoV-2 antigen and confirmed infection using an RT-qPCR and measured IgM and IgG antibodies in fully vaccinated participants from municipalities with higher and lower vaccination rates. Results: We showed that participants from the least vaccinated municipalities were more likely to have detectable IgM antibodies and a positive antigen/RT-qPCR result for SARS-CoV-2 than participants from the most vaccinated municipalities. There were no differences between the vaccines used (BNT162b2, Ad26.COV2.S, AZD1222, and CoronaVac) and antibody production. Conclusions: Our study evaluated municipal vaccination coverage and its effects on mortality, infections, and anti-SARS-CoV-2 antibodies during a critical phase of the pandemic. The results suggest that higher vaccination coverage reduces acute cases and confers higher memory antibody levels against SARS-CoV-2. Even with a full vaccination schedule, individuals living in places with low vaccination rates are more susceptible to infection.

## 1. Introduction

SARS-CoV-2 is a viral infectious agent that caused the Coronavirus Disease 2019 (COVID-19) pandemic, which has affected several countries and millions of people. This virus is of great interest for epidemiological studies due to its highly variable nature, resulting in the emergence of variants that are more pathogenic, are more transmissible, and escape the mechanisms of adaptive immunity [1]. In Brazil, during the global pandemic, the main variants responsible for COVID-19 outbreaks were the P.1 (Gamma), B.1.617.2 (Delta), and B.1.1.529 (Omicron) variants. The latter was reported to the WHO on 24 November 2021 and, after just two days, was declared a variant of concern (VOC) [2]. The many mutations in this variant, compared to the original Wuhan strain, make it more capable of evading the immune system than others [3]. In December 2021, the variant was already circulating in Brazil [4]. Due to this new strain of the virus, which caused the third wave of the pandemic [5], the WHO reported in the penultimate week of January 2022 the highest incidence of cases per week since the beginning of the pandemic and, despite vaccination, the number of deaths recorded exceeded the previous ones [2,6]. In Brazil, in the last week of January 2022, more than 1 million cases and 3000 deaths were reported [6]. In the same week, in Sergipe, a state located in northeastern Brazil and where our study was carried out, more than 7000 cumulative cases and 17 deaths were recorded [7].

The emergence of more pathogenic and infectious variants of SARS-CoV-2 is a significant concern for global authorities as it prevents the easing of contingency and mitigation measures that affect the economy and development of several countries in the globalized world [8]. Vaccines against COVID-19 can target the particularities of SARS-CoV-2 mutations and guarantee better immune responses to infection by different variants [9]. Although vaccination does not prevent viral spread, without control and the vaccination coverage of a population, it is not possible to contain the arrival and outbreaks of new variants [10]. The WHO has created a strategy for the global community to reach at least 70% vaccination coverage by mid-2022 [11]. In Brazil, to control SARS-CoV-2 and its variants, a vaccination campaign against COVID-19 began in January 2021 after vaccines were approved by ANVISA (National Health Surveillance Agency) and made available to the population [12]. The vaccination schedule proposed by the Ministry of Health and the National Immunization Program allowed the first doses of the CoronaVac (Sinovac^®^ Biotech, Beijing, China), BNT162b2 (BioNTech^®^, Mainz, Germany), or AZD1222 (Oxford University, Oxford, UK) and Ad26.COV2.S (Johnson&Johnson^®^, New Brunswick, NJ, USA) vaccines to be administered [12]. In the same year, the northeastern region of the country had a total of around 86,000 doses administered to its population, and up to the time of this study, the State of Sergipe, to which 75 cities belong, presents around 5.5 million doses applied [13,14,15].

High vaccination rates are of paramount importance to preventing severe disease and deaths, especially at the height of a pandemic, as was, in this case, caused by the B.1.1.529 variant [16]. However, vaccination is not adhered to equally in Brazilian municipalities, and more vaccinated municipalities have lower rates of cases and deaths from COVID-19 [17]. In this context, some places suffer a more significant impact than others since several factors, such as vaccination, the movement of people, and adherence to preventive measures, are directly involved in viral propagation. Moreover, this population, especially in the context of the emergence of new variants, may be subject to substantial increases in cases and deaths. Given this, we studied the circulation of SARS-CoV-2 and the response to vaccination in the least and most vaccinated municipalities during the Omicron wave in the Brazilian State of Sergipe.

## 2. Materials and Methods

### 2.1. Study Design and Target Public Selection

**Figure 1:**
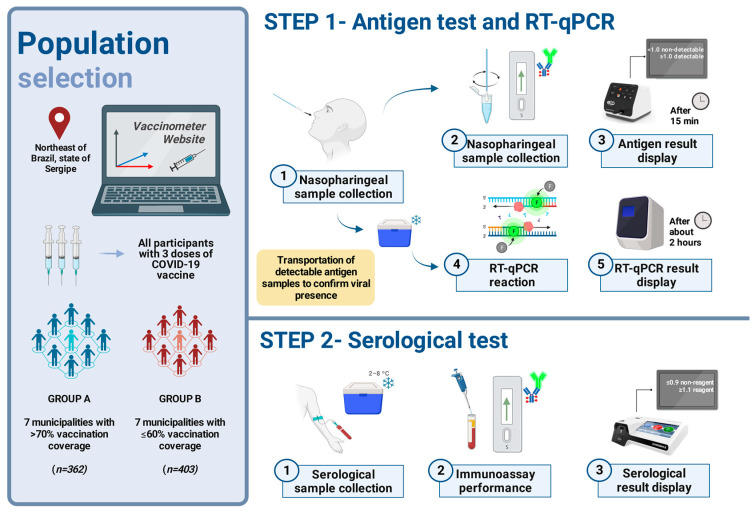
Study workflow.

**Figure 2:**
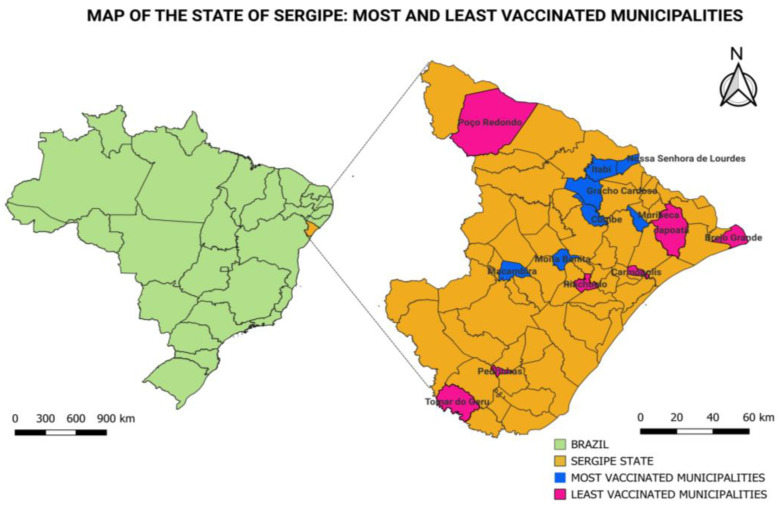
Maps of Brazil and the State of Sergipe with the most and least vaccinated municipalities.

The study methodology is demonstrated in Figure 1. This cross-sectional study was approved by the National Bioethics Committee of Brazil (Certificate of Presentation of Ethical Appreciation 31018520.0.0000.5546). For the selection of the fourteen municipalities included in our study, we analyzed data released by the Brazilian vaccinometer (Vacinômetro COVID-19) from the National Health Council website (https://conselho.saude.gov.br/vacinometro (accessed on 7 January 2022)) [15] (Figure 1, Step A). This website was created to disseminate updates on the vaccination status of the population of each state and municipality of Brazil. We ranked the most vaccinated municipalities as those that had vaccination coverage of 70% or more. We found that the seven most vaccinated municipalities in Sergipe (Group A) at the time of this study were *Graccho Cardoso, Cumbe, Moita Bonita, Muribeca, Macambira, Itabi*, and *Nossa Senhora de Lourdes*. The municipalities of *Carmópolis, Poço Redondo, Tomar do Geru, Riachuelo, Brejo Grande, Pedrinhas*, and *Japoatã* were the places (Group B) that had the lowest vaccination rates of the populationy (Figure 2). All of these have 60% or lower vaccination coverage. The software QGIS^®^ Version 3.32.1 [18] was used to represent these cities and the State of Sergipe, Brazil, with cartographic bases from the IBGE (Brazilian Institute of Geography and Statistics) [19]. The results obtained in our study were reported by the Municipal Health Secretariats and then fed into the database of notifications of COVID-19 cases in the state [20].

### 2.2. Data Collection

This study occurred from 11 February to 25 March 2022 and included the participation of seven hundred and sixty-five (765) individuals. As inclusion criteria for this study, participants must have received the booster dose (third dose) of the vaccine, live in one of the cities in question, and consent to participate. First, the participants answered a questionnaire to collect personal data such as their name, sex, age, hometown, vaccines used, and the number of doses. The composition of the study population predominantly reflected an asymptomatic condition, estimated at approximately 80%, as documented in previous publications by our research group [21,22,23]. A multi-stage sampling approach was adopted, using a cluster selection method that incorporated a combination of simple random sampling. The stratification included representatives of different socioeconomic strata as well as different social and occupational groups. Subsequently, these individuals were directed to the collection of samples for testing based on the protocols of studies already published by our group [21,22,23,24,25,26,27].

### 2.3. Antigenic and RT-qPCR Detection Process

After obtaining informed consent, participants underwent the ECO F COVID-19 Ag^®^ test (https://ecodiagnostica.com.br/diagnostico-rapido/eco-f-covid-19-ag/ (accessed on 27 December 2023)) [28] to detect the SARS-CoV-2 nucleocapsid (N) protein, providing quantitative results with a specificity (to date) and sensitivity (to date) of >99.9% and 96.52% (symptomatic) and 96.6% (asymptomatic), respectively. Confirmation of the presence of SARS-CoV-2 was achieved through the Applied Biosystems™ TaqPath™ COVID-19 CE-IVD RT-PCR Kit (Foster City, CA, USA), developed by Thermo Fisher^®^ (Waltham, MA, USA) (https://www.thermofisher.com/order/catalog/product/A48067?SID=srch-srp-A48067 (accessed on24 August 2023)) [29], exhibiting 100% specificity and sensitivity (https://assets.thermofisher.com/TFS-Assets/GSD/Reference-Materials/taqpath-ceivd-rt-pcr-kit-technical-bulletin.pdf (accessed on 24 August 2023)) [30]. Swab samples were stored in viral transport media and processed using the QuantStudio™ 5 Dx Real-Time PCR instrument, with the results released to participants within approximately two days.

### 2.4. Antibodies Detection Process

Blood samples for the serological analysis of IgM and IgG antibodies against SARS-CoV-2 were collected in serum separator gel tubes, stored in thermal boxes at 2–8 °C, and transported for further analysis. This investigation utilized the Fluorescence-based Lateral Flow Immunoassay method (ichroma™ II COVID-19 Ab) with an Ichroma™ II Reader from Boditech Med Inc., Gangwon, Republic of Korea (https://www.boditech.co.kr/en/product/instruments/id/4 (accessed on 7 August 2023)) [31], which interprets results as reactive (≥1.1), non-reactive (≤0.9), or indeterminate (0.9–1.1). This method demonstrates a specificity of 97.0% and a sensitivity of 95.8% [31,32].

### 2.5. Statistical Analysis and Data Visualization

The results were analyzed using IBM^®^ SPSS^®^ Statistics software (version 26.0 for Windows) [33]. Descriptive statistics were determined. Normality tests were conducted considering *p* > 0.05 for inhomogeneous samples [34]. Chi-square tests of independence (2 × 2), Fisher’s exact test, and prevalence ratio and odds ratio analyses were carried out [35,36,37,38]. To assess the significance of these tests, a *p*-value < 0.05 was considered statistically significant and, where appropriate, a 95% confidence interval was applied [39]. We used the Circos^®^ graph (http://circos.ca/ (accessed on 13 August 2023)) [40] to visualize the distribution of vaccine combinations in the most and least vaccinated municipalities and to assess IgG antibody distribution with these combinations.

## 3. Results

### 3.1. Data Analysis in the Tested Municipalities

Of the 75 municipalities in the State of Sergipe, Brazil, during the test period, seven municipalities vaccinated most of their estimated populations (>70%), and another seven municipalities did not demonstrate satisfactory adherence to the vaccine program compared to the other municipalities in the state (≤60%), as described in Table 1 [20,41,42]. For this reason, these 14 municipalities were selected.

During data collection, we made an initial estimate of the sample size, taking into account the vaccinated population. As the tests were carried out at different times in the municipalities, depending on the adherence of participants who met the inclusion criteria, we calculated the standard error using the result of the sample size estimate of the vaccinated population with the sample size actually collected (Table 1). This allowed us to show the variation between the expected average and the actual sample size collected. Therefore, the groups did not have a homogeneous number of participants. Of the 765 study participants, 47.3% (*n* = 362) were residents of the most vaccinated municipalities (group A), and 52.7% (*n* = 403) were residents of the least vaccinated municipalities (group B).

Of the participants from the most vaccinated towns (group A), 237 were female and 125 were male. The average age was 40.90 years (SD = 17.249; 95%CI = 39.11–42.68). Of the participants from the least vaccinated towns (group B), 218 were female and 185 were male. The average age was 44.37 (SD = 15.842; 95%CI = 42.82–45.92). Overall, the majority of the participants were women (*n* = 455) compared to men (*n* = 310).

Mortality rates in the municipalities, presented in Supplementary Table S4, are publicly available data and provide context for our study [42]. The mortality rate for each municipality was calculated using the number of COVID-19-related deaths by the end of the study, corrected for the size of the population in each municipality (Supplementary Table S4). Examining the descriptive data reveals that the municipalities with the lowest vaccination rates exhibited a higher mortality rate score (M = 1.84; SD = 0.62) compared to the most vaccinated municipalities (M = 1.32; SD = 0.46). An effect size analysis was carried out to check the strength of the relationship between the variables, although no significance was found. We found that this relationship has a high effect size (Cohen’s d = 1.01).

### 3.2. Association between Antigen/RT-qPCR and IgM Antibodies in the Tested Municipalities

A chi-square test of independence (2 × 2) was performed to investigate whether there was an association between the municipalities tested (group A and group B) and antigen/RT-qPCR (detectable and non-detectable) and IgM antibody (reactive and non-reactive). The association between the tested municipalities and the antigen/RT-qPCR (χ^2^(1) = 1.945, *p* > 0.05; Φ = 0.05) (Supplementary Tables S1–S3) did not show a statistically significant association. A higher prevalence ratio was observed in group B residents (66.7%; *n* = 16) compared to group A residents (33.3%; *n* = 8) for the presence of SARS-CoV-2 detected via antigen/RT-qPCR. A statistically significant association was found between tested municipalities and IgM antibodies (χ^2^(1) = 12.076, *p* < 0.001; Φ = 0.126) (Supplementary Table S3). An odds ratio analysis demonstrated that individuals living in municipalities with a low vaccination rate (group B) were 2.89 (z = 3.34; 95%CI = 1.5518 to 5.3896; *p* = 0.0008) times more likely to have a reactive IgM antibody when compared to individuals living in municipalities with a high vaccination rate (group A).

### 3.3. Distribution and Combinations of Vaccines, Together with the Production of IgG Antibodies, in the Most and Least Vaccinated Municipalities

**Figure 3:**
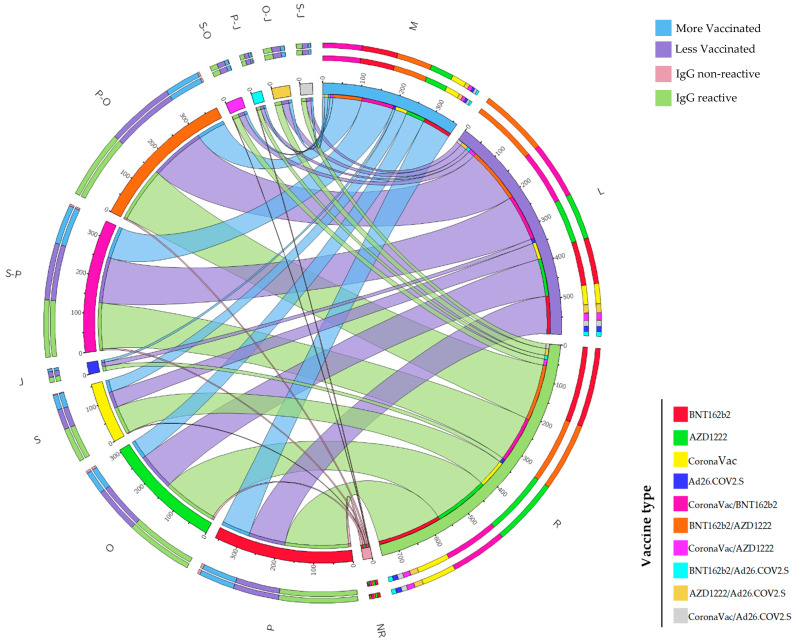
List of combinations of vaccines administered in the more vaccinated (M) and less vaccinated (L) municipalities, with the distribution of vaccines related to IgG antibodies. Regarding the types and quantities of vaccines, we obtained P (BNT162b2; M = 79, L = 101), O (AZD1222; M = 51, L = 102), S (CoronaVac; M = 33, L = 45), J (Ad26.COV2. S; M = 6, L = 6), S-P (CoronaVac/BNT162b2; M = 87, L = 42), P-O (BNT162b2/AZD1222; M = 78, L = 73), S-O (CoronaVac/AZD1222; M = 8, L = 10), P-J (BNT162b2/Ad26. COV2.S; M = 5, L = 7), O-J (AZD1222/Ad26.COV2.S; M = 9, L = 10), and S-J (CoronaVac/Ad26.COV2.S; M = 6, L = 7). When considering the reactive IgG antibody (R) and the non-reactive IgG antibody (NR), we obtained the following results: P (R = 174, NR = 6), O (R = 148, NR = 5), S (R = 76, NR = 2), J (R = 12, NR = 0), S-P (R = 125, NR = 4), P-O (R = 150, NR = 4), S-O (R = 17, NR = 1), P-J (R = 10, NR = 2), O-J (R = 19, NR = 0), and S-J (R = 13, NR = 0).

During the questionnaire, participants were asked which immunizers administered the vaccines. Of the four vaccines distributed at the time in Brazil (BNT162b2, AZD1222, CoronaVac, Ad26.COV2.S) (Supplementary Tables S1 and S2), BNT162b2 was the most frequently administered among the participants with *n* = 180 (group A = 79; group B = 101), followed by AZD1222 with n = 153 (group A = 51; group B = 102) (Figure 3). In the cities tested, many participants reported having received different types of vaccines, which resulted in several different heterologous combinations. Of the heterologous combinations, we obtained six combinations (CoronaVac/BNT162b2, BNT162b2/AZD1222, CoronaVac/AZD1222, BNT162b2/Ad26.COV2.S, AZD1222/Ad26.COV2.S, and Corona-Vac/Ad26.COV2.S). Fisher’s exact test showed no difference in the distribution of the different combinations of IgG antibody vaccines (*p* > 0.05). Although we now know that BNT162b2, compared to the other vaccines distributed at the time, has a more significant potential to produce neutralizing antibodies, combining different vaccines proved effective in producing neutralizing antibodies against the variants in force [43]. The chi-square test of independence (2 × 2) showed that there was a difference between the distribution of anti-tibial IgG antibodies in the municipalities tested (χ^2^(1) = 9.453, *p* < 0.01; Φ = 0.11). The odds ratio showed that residents of the municipalities with the lowest vaccination rates were 5.60 (z = 2.742; 95%CI = 1.6342 to 19.1546; *p* = 0.0061) times more likely to present non-reactive IgG antibodies (18/403) compared to residents of the most vaccinated municipalities (3/362). We suggest that in a population with higher vaccination coverage, the chances of producing neutralizing antibodies are higher than in a population with low vaccination coverage. However, we did not test for neutralizing antibodies or cellular immune responses, T cells, against the coronavirus, which also play a role in protection [44,45,46].

## 4. Discussion

Our study evaluated the populations of the municipalities with the lowest and highest vaccination rates in a Brazilian state. We performed antigen testing with a confirmatory RT-qPCR for viral detection and IgM and IgG antibody tests. In addition, we investigated the differences in mortality and case prevalence in these municipalities. We observed, by the size of the effect, that the more vaccinated municipalities have a lower mortality rate compared to the municipalities with lower vaccination coverage. A study carried out in the United States [17] evaluated less and more vaccinated municipalities and found that mortality and the number of cases were higher in less vaccinated locations during the outbreak of the Delta variant. Based on this study and our results, it can be assumed that vaccination at the community level promotes a significant response against the COVID-19 virus, which may also explain the risks of the less vaccinated population showing positive results for antigen/RT-qPCR and IgM tests that are correlated with the presence of viral infection [47].

Furthermore, the increase in the IgM antibody, which is present in acute or recent infections, produced on average seven days after the onset of symptoms [48], may be associated with the presence of a more significant number of unvaccinated individuals in group B (municipalities with a lower vaccination rate), a fact that may increase the chances of the virus circulating, through contamination via aerosols, surfaces, and direct contact in the case of family members of infected individuals [49]. This increase in unvaccinated individuals, as outlined by previous publications by our team [50], highlights a significant challenge in Brazil related to the effects of anti-vaccine ideas among the less educated and culturally disadvantaged population. A Brazilian study [51] identified high levels of vaccine hesitancy related to COVID-19 associated with behavioral factors such as little fear of the disease and a belief in the unnecessariness of the vaccine for those who have already had an infection. The dissemination of these anti-vaccine concepts by science deniers through social media platforms contributes substantially to the reluctance to seek vaccination. Therefore, we suggest that individuals living in places with populations with low vaccination coverage have a high risk of infection, even if they have complied with the vaccination schedule. We emphasize the importance of community vaccination as a determining factor in preventing contamination, highlighting the importance of effective public policies, advertising campaigns, incentives for home vaccination, and active search strategies to address this obstacle and ensure broad vaccination coverage in these vulnerable communities.

In our study, we found that all the vaccines used by the Brazilian public (BNT162b2, AZD1222, Ad26.COV2.S and CoronaVac) can promote an antibody response (Figure 3). However, this response was not measured in a quantitative result for a better comparison of the vaccines. Our data may suggest that the booster dose helped to further control the impact of the circulating variant in the State of Sergipe, Brazil, where we observed a low detectable antigen/RT-qPCR count in Groups A and B (Supplementary Table S3). In addition, bivalent vaccines are currently being applied for the booster dose in the Brazilian population. These vaccines are more effective at reducing deaths and hospitalizations compared to monovalent boosters, which were the types of booster we evaluated in our study since the bivalent vaccine had not yet been made available to the Brazilian public at the time the study took place [52,53]. However, more studies are needed to assess the difference in immune response to bivalent and monovalent boosters.

This study has some limitations. Although we calculated the sample size, we were unable to reach the expected number of participants in our study because the majority of the population had already been vaccinated and were less likely to seek testing. In addition, there was fear on the part of the population in the municipalities of taking the test and discovering a possible infection. In addition, we did not perform an ELISA immunoassay or a neutralizing antibody test, which are considered gold-standard tests for assessing antibody immunity.

In summary, in our study, we evaluated the impact of vaccination coverage on mortality, the presence of infections, and anti-SARS-CoV-2 antibodies at the municipal level during the worst phase of the COVID-19 pandemic. Thus, it is possible to know the impact of COVID-19 in municipalities with lower and higher rates of vaccination coverage, inferring that even if an individual is fully vaccinated, the risk of infection may be higher in places with low vaccination coverage. We also evaluated the different vaccines used in Brazil, either by brand or by homologous and heterologous scheme, and their effectiveness in the prevalence of IgM and IgG anti-SARS-CoV-2 antibodies. Therefore, our findings allow government interventions to intensify the application of vaccines and the screening of active cases, seeking to interrupt the chain of transmission, especially in municipalities with a higher mortality rate.

## Figures and Tables

**Table 1:** Tested populations of the most and least vaccinated municipalities by epidemiological ranking.

	Population Estimation *n* *	Vaccinated *n* (%) **	Tested Residents *n* (%) ***
Group A			
Gracho Cardoso	5.818	4.538 (78)	50 (12.32)
Cumbe	3.987	3.070 (77)	61 (10.76)
Moita Bonita	11.335	8.728 (77)	39 (14.39)
Muribeca	7.625	5.719 (75)	43 (13.54)
Macambira	6.919	5.189 (75)	65 (10.36)
Itabi	4.903	3.677 (75)	57 (11.29)
Nossa Senhora de Lourdes	6.483	4.862 (75)	47 (12.81)
Group B			
Japoatã	13.434	8.060 (60)	54 (11.76)
Pedrinhas	9.602	5.665 (59)	34 (15.58)
Brejo Grande	8.309	4.902 (59)	71 (9.74)
Riachuelo	10.213	5.923 (58)	51 (12.18)
Tomar do Geru	13.536	7.715 (57)	66 (10.28)
Poço Redondo	34.775	19.822 (57)	51 (12.22)
Carmópolis	16.634	9.315 (56)	77 (9.22)

* Population numbers for each municipality derived from estimates provided by the Sergipe State Secretariat. ** Data taken from the epidemiological bulletin issued by each municipality show the number of individuals vaccinated and the percentage related to the total estimated population of each municipality. *** Number of people tested in each municipality and the percentage of the standard error. This calculation focused explicitly on the vaccinated population of the individuals who were tested, incorporating a 95% confidence interval for statistical robustness.

## Data Availability

If you are interested in further data on the results, please contact the corresponding author.

## Supplementary Materials

The following supporting information can be downloaded at https://www.mdpi.com/article/10.3390/life14010094/s1, Table S1: Demographic and clinical data—most vaccinated municipalities; Table S2: Demographic and clinical data—least vaccinated municipalities; Table S3: Municipalities tested vs Antigen/RT-qPCR and IgM; Table S4 Number of deaths from the start of vaccination until the end of March 2022 and results of the mortality rate.
